# Red blood cell folate level is associated with periodontitis in American adults: results from the NHANES 2009–2014

**DOI:** 10.1186/s12903-024-04599-7

**Published:** 2024-07-21

**Authors:** Zefei Liu, Shiyi Luo, Ruofeng Jiao, Wei Li, Fuqian Jin, Jiangling Sun, Shu Ma, Jukun Song, Zhu Chen

**Affiliations:** 1 School of Stomatology, Zunyi Medical University, Zunyi, Guizhou Province 563000 China; 2 Guizhou University Medical College, Guiyang, Guizhou Province 550025 China; 3 Guiyang Stomatological Hospital, Guiyang, Guizhou Province 550005 China; 4 Oral & Maxillofacial Surgery Department, Guizhou Medical University, Guiyang, Guizhou Province 550001 China

**Keywords:** Periodontitis, Red blood cell folate, NHANES database

## Abstract

**Background:**

Red blood cell (RBC) folate is an indicator of long-term folate nutrition. Whether there is an association between RBC folate and periodontitis is unclear. This study aimed to use the NHANES database to determine whether RBC folate is associated with moderate/severe periodontitis.

**Methods:**

A cross-sectional analysis of 10,151 participants in the NHANES database from 2009 to 2014 was performed. Multivariate logistic regression was used to analyze the independent relationship between RBC folate and moderate/severe periodontitis. The generalized additive model (GAM), restricted cubic splines (RCS), smooth curve fitting, and threshold effect analysis were used to explore the dose–response relationship and the potential nonlinear relationship between RBC folate and periodontitis. Finally, subgroup analysis and interaction tests were performed to determine the effect of covariates on the relationship between RBC folate and moderate/severe periodontitis.

**Results:**

After adjusting for all confounders, there was a negative association between RBC folate concentration and moderate/severe periodontitis. The lowest fraction Q1 (< 360 ng/mL) of RBC folate concentration was used as the reference group, multivariable-adjusted ORs and 95% CIs of the second (360-463 ng/mL), third (464-569 ng/mL), fourth (570-732 ng/mL), and the highest quintile (> 733 ng/mL) categories were 0.88 (0.77, 1.01), 0.83 (0.72, 0.96), 0.77 (0.67, 0.90), 0.65 (0.56, 0.77) respectively. Additionally, a threshold nonlinear association was found between RBC folate (ng/mL) log2 transformation and moderate/severe periodontitis.

**Conclusion:**

This cross-sectional study revealed a negative relationship between RBC folate and moderate/severe periodontitis within a certain threshold range. Dentists and policymakers should pay closer attention to oral hygiene and health care for people with low or high RBC folate levels. Further causal and longitudinal research mechanisms are needed to validate our findings.

**Supplementary Information:**

The online version contains supplementary material available at 10.1186/s12903-024-04599-7.

## Introduction

Periodontitis is a chronic multifactorial inflammatory disease associated with plaque biofilms, local stimuli, destructive immune response, personal lifestyle, and some systemic factors [1, 2] and leads to local and systemic chronic inflammatory reactions [3]. Persistent periodontal inflammation can destroy periodontal supportive tissues, leading to alveolar bone loss and tooth loss [4]. The National Health and Nutrition Examination Survey (NHANES 2009 to 2012) reported that about 50% of American adults over the age of 30 have periodontitis [5]. The overall prevalence of periodontitis is 45% to 50%, with the most severe forms of periodontitis affecting 11.2% of the world's population [6]. It's obvious that moderate/severe periodontitis is increasingly becoming a serious problem and reducing our quality of life.

Recent studies have shown an association between periodontal health and nutritional status [7]. Folate, a water-soluble B vitamin found in grains and green vegetables, participates in various metabolic pathways, playing a crucial role in cell division and DNA synthesis [8–10]. Folate is necessary to maintain and produce new cells during periodontal tissue development and healing [11].

Folate undergoes a series of metabolisms culminating in the conversion to 5-MethylTHF which is stored in serum and red blood cells [12].

There have been previous reports on the relationship between folate and periodontitis, but there is still some controversy [13]. A randomized clinical controlled trial showed that systemic folate intake adjunctive to periodontitis treatment can improve periodontal clinical indicators and biochemical parameters [14]. In cross-sectional studies, lower folate intake and a higher risk of periodontitis were associated [15, 16] and the average serum folate level in the periodontitis group was lower than in healthy volunteers [17]. Inadequate folate intake may lead to elevated plasma homocysteine concentrations [18], further increasing the risk of periodontitis [19, 20]. However, in a recent dataset survey (Using the Big Mouth Repository), folate supplementation has been associated with an increased risk of periodontitis [21]. Additionally, some scholars have shown that folate receptor (FOLR1) does not appear to have a role in the detection of periodontal disease [11]. Previous studies indicated that low serum folate levels in older adults were independently associated with periodontal disease (from NHANES 2001 to 2002) [22]. However, serum folate only reflects the recent folate nutritional status, and the single indicator of "low serum folate" cannot distinguish between a short-term situation caused by transient insufficient dietary folate intake and a chronic folate deficiency state [21]. Red blood cell folate (RBC folate) level can reflect chronic or long-term (within 4 months) folate nutrition and is generally used to evaluate the effect of folic acid supplementation and correction of deficiency [23, 24]. The literature states that the most accurate measurement of folate should be red blood cell folate measurement, followed by serum folate measurement [25].

Due to this, research on the relationship between RBC folate and periodontitis in a large and representative population is necessary to develop. The study aimed to use the National Health and Nutrition Examination (NHANES) to study the association of RBC folate with moderate/severe periodontitis. Exploring the relationship between RBC folate and periodontitis can help dentists and policymakers better understand the pathogenesis of the disease and provide more effective treatment and prevention strategies to improve people's oral health.

## Methods

### Data source and study population

The data on exposure and outcome were collected from the three continuous National Health and Nutrition Examination Survey (NHANES) (https://www.cdc.gov/nchs/nhanes) cycles from 2009–2014. NHANES is run by the National Center for Health Statistics (NCHS), which is part of the Centers for Health Statistics (NCHS). NHANES is a nationally representative survey of nutrition and health in the United States, with data obtained from health interviews at participants' homes, health exams at mobile testing centers (MECs), and laboratory specimens. NHANES' ethical review has passed the National Center for Health Statistics Research Ethics Review Board, so this manuscript does not require additional ethical review [26].

This study initially included 30,468 participants (NHANES 2009 to 2014), which has the most recent periodontal examination data in American adults. The inclusion criteria were as follows: (1) NHANES participants aged 30 years and older; (2) subjects received oral periodontal examination; (3) NHANES participants with red blood cell folate data. A very small number of participants lacked covariates such as education level, smoking consumption, and body mass index (BMI) were excluded. Finally, 10,151 participants were included in the analysis.

### Definition of periodontitis

Exploration of periodontal pockets probing depth (PD), and loss of clinical attachment (AL) are common indicators for assessing the severity of periodontitis [27]. This study used CDC/AAP (CDC, Centers for Disease Control and Prevention; AAP, American Academy of Periodontology) periodontitis classification criteria proposed by Eke et al. in 2012 [28]. Mild periodontitis was defined as 2 interproximal sites with AL ≥ 3 mm, and ≥ 2 interproximal sites with PD ≥ 4 mm (not on the same tooth) or one site with PD ≥ 5 mm. Moderate periodontitis was defined as 2 interproximal sites with AL ≥ 4 mm (not on the same tooth), or ≥ 2 interproximal sites with PD ≥ 5 mm (not on the same tooth). Severe periodontitis was defined as 2 interproximal sites with AL ≥ 6 mm (not on the same tooth) and ≥ 1 interproximal site with PD ≥ 5 mm. The outcome variable (binary variable) was classified as no/mild periodontitis and moderate/severe periodontitis [29].

In the mobile examination center, periodontists probed all 28 teeth of the participants who met the requirements (six sites per tooth) but excluded the third molar [30].

To ensure the credibility of the data, the mobile inspection center has conducted professional training and strict calibration. Detailed information on the specific training and calibration process can be found in the NHANES 2000 Oral Health Training Manual [31].

### Red blood cell folate

Whole blood and blood serum were processed, stored, and shipped to the Division of Laboratory Sciences, National Center for Environmental Health for analysis. After adjusting the RBC volume and serum folate, RBC folate concentration was calculated from whole blood folate by microbiological assay (MA) method using Lactobacillus rhamnosus/Lactobacillus casei. Compared to the Bio-Rad (BR) Quanta Phase II radio assay (before NHANES 2007), NHANES recommended MA as the more accurate gold standard. Impossible values were sent to the laboratory for review and confirmation. When analyzing RBC folate concentrations as a continuous variable, a log2 transformation was applied to meet a normal distribution [32].

### Confounding factors

**Fig. 1:**
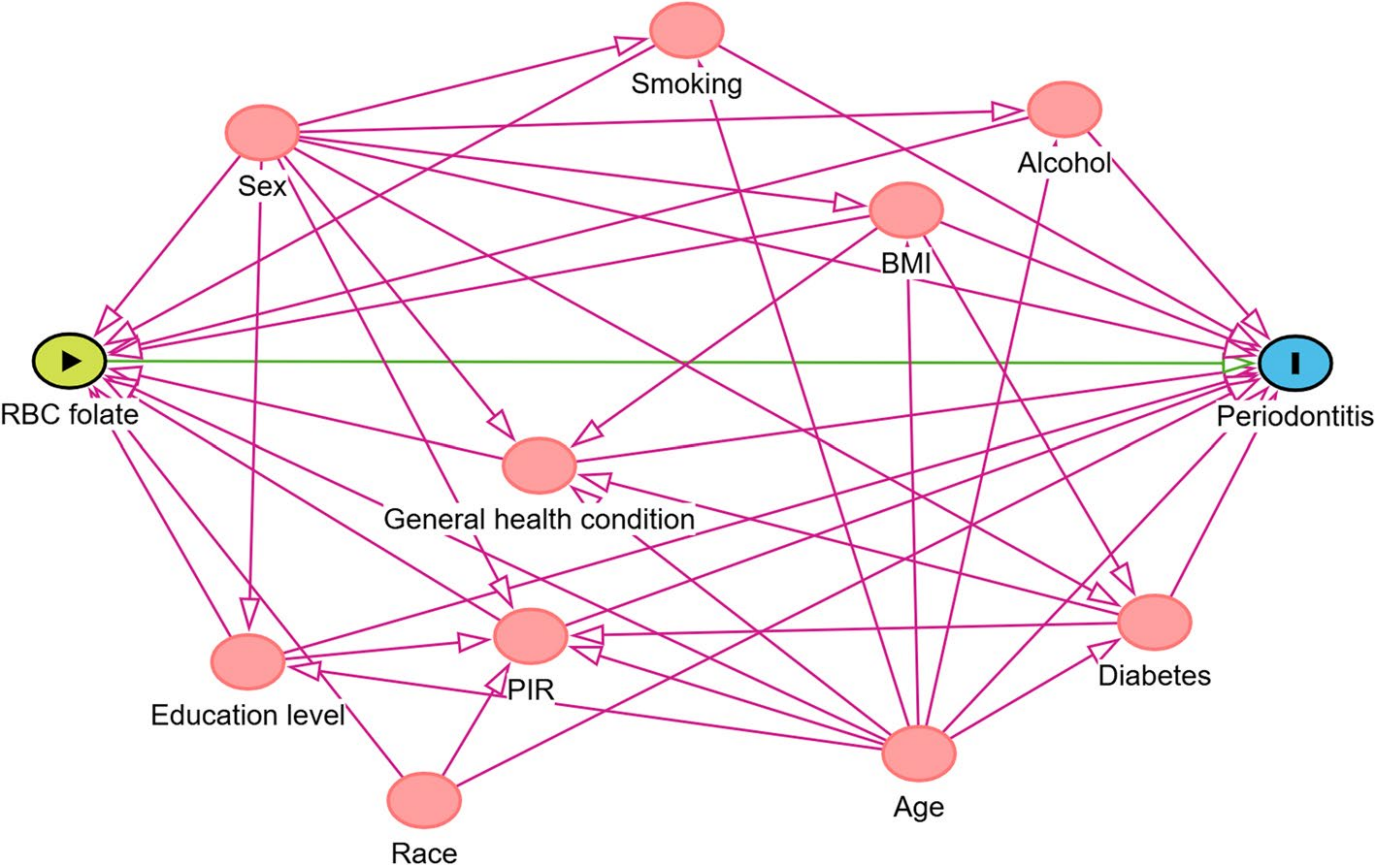
Directed acyclic graph (DAG) of the association between RBC folate, periodontitis, and all confounding factors

The selection of covariates was largely informed by previous research. The confounding factor was defined as covariates affecting both RBC folate concentration and periodontitis [33]. We comprehensively screened age, sex, ethnicity, education level (< = high school, > high school), poverty income ratio (PIR: low 0–1.56, middle 1.57–3.62, high 3.63–5) [34], body mass index (BMI), smoking consumption, alcohol consumption, general health condition, and diabetes as potential confounding factors [34–38]. We draw a directed acyclic graph (DAG) to clarify the association between exposure, outcome, and all confounding factors (Fig. 1). Based on WHO recommendations, we classify BMI as underweight to normal weight (< 24.9 kg/m^2^), overweight (25.0–29.9 kg/m^2^), or obese (> = 30.0 kg/m^2^) [34]. Smoking consumption was classified into three levels: smoked less than 100 cigarettes in life as ‘never’, smoked more than 100 cigarettes in life and smoked not at all now as ‘former’, smoked more than 100 cigarettes in life and smoked some days or every day is defined as ‘current’) [39]. The National Institute on Alcohol Abuse and Alcoholism (NIAAA) classified alcohol consumption as follows: none, moderate (1 drink per day for women and 1–2 drinks per day for men), heavy (2–3 drinks per day for women and 3–4 drinks per day for men), and binge (4 drinks per day for women and 5 drinks per day for men) [40]. We divided general health conditions into two categories: excellent/very good/good and fair/poor [34]. Diabetes was defined as oral glucose tolerance test /OGTT (fasting blood glucose > 7.0 mmol/L, 2-h postprandial blood glucose > 11.1 mmol/L), glycated hemoglobin A1c (HbA1c) > 6.5% and self-reported doctor diagnosis of diabetes [41].

### Statistical analysis

**Fig. 2:**
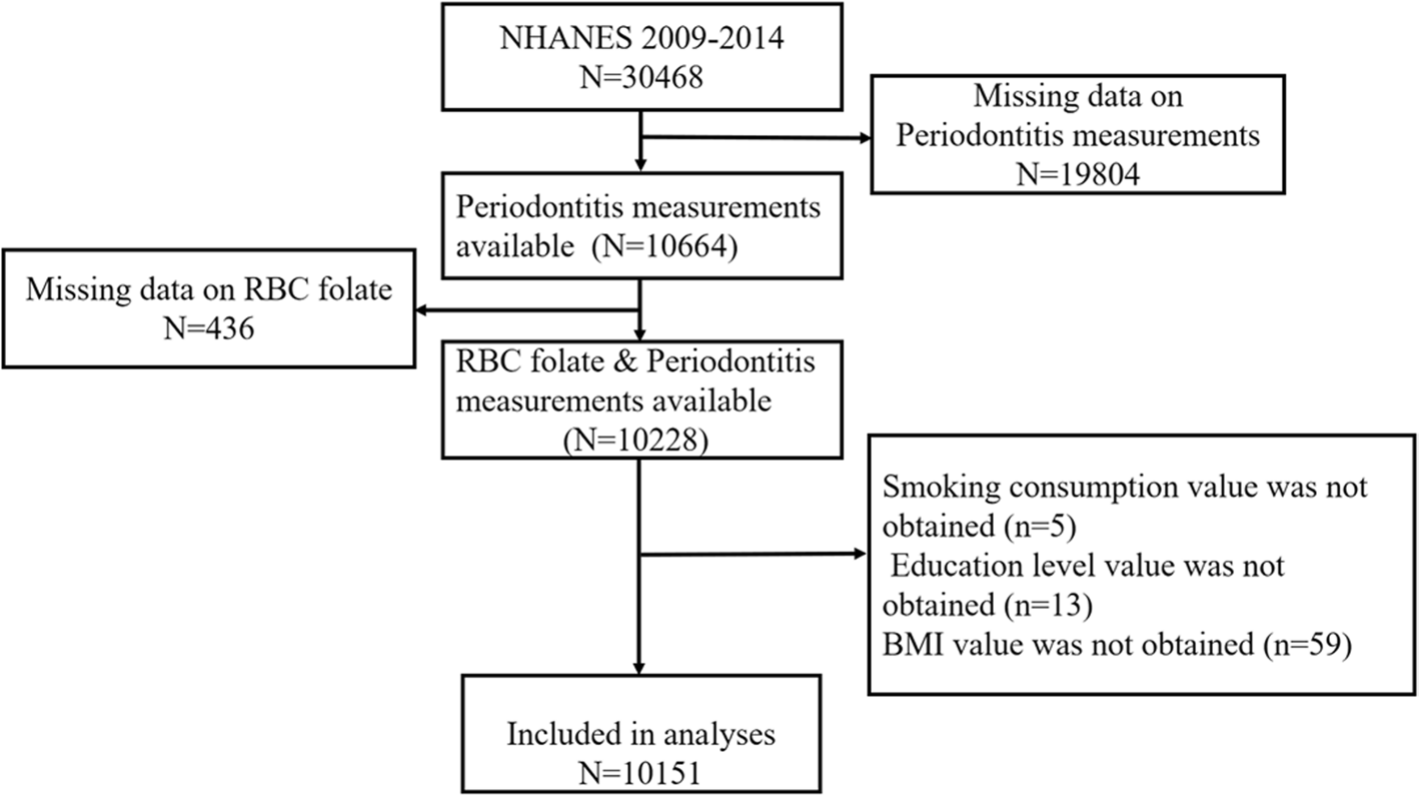
Flowchart of participants selection

The study used Empower Stats 4.1(http://www.empowerstats.com) combined with R packages (R4.2.1 http://www.R-project.org) for data analyses. The data screening process is shown in Fig. 2. The Kruskal–Wallis H test (continuous variable) and chi-square test (categorical variable) were used to estimate whether there is a statistical difference between different RBC folate groups (quintiles) [34]. Under the two-sided test, *p* < 0.05 is considered statistically significant. Multivariate logistic regression was used to analyze the independent relationship between RBC folate and moderate/severe periodontitis (unadjusted model; model 1 adjusted for age, sex, and ethnicity; model 2 adjusted for sex, age, race, educational level, PIR, BMI, alcohol consumption, smoking consumption, general health condition, and diabetes). The generalized additive model (GAM), restricted cubic splines (RCS), smooth curve fitting, and threshold effect analysis were used to explore the dose–response relationship and the potential nonlinear relationship between RBC folate and moderate/severe periodontitis [34]. Trend *p* values were used as sensitivity analysis to ensure the robustness of the results [34]. Finally, subgroup analysis and interaction tests were performed to determine the role of covariates between RBC folate and periodontitis [40].

## Results

### Baseline characteristics of the population

The baseline characteristics of the study population are presented in Table 1. A total of 10,151 participants were included in the analysis. The study divided RBC folate concentration into five groups. Continuous variables are presented as mean ± standard deviation, and categorical variables are presented as percentages. Overall, participants with high levels of RBC folate were more likely to be older, female, non-Hispanic white, and individuals with more than a high school education. As RBC folate concentrations increased, the proportion of “never” smokers and “former” smokers gradually increased, while “current” smokers gradually decreased. Interestingly, the proportion of moderate alcohol consumers also increased with increasing RBC folate levels. In addition, participants with lower RBC folate typically had poorer periodontal clinical parameters such as mean PD, mean CAL, extent PD ≥ 4 mm (%), etc.”.

**Table 1:** Baseline characteristics of participants (*N* = 10,151)

Characteristics	Quintile categories of red blood cell folate levels, ng/mL					*p* values
	Q1 (< 360)	Q2(360–463)	Q3(464–569)	Q4(570–732)	Q5 (> 733)	
*N*	2542	2309	2001	1792	1507	
Age (years, mean ± SD)	48.9 ± 13.2	49.9 ± 13.1	51.3 ± 13.7	53.7 ± 14.4	59.2 ± 15.1	< 0.001
Age group						< 0.001
< 60	1937 (76.2%)	1697(73.5%)	1388(69.4%)	1124(62.7%)	736 (48.8%)	
≥ 60	605 (23.8%)	612 (26.5%)	613 (30.6%)	668 (37.3%)	771 (51.2%)	
Sex						< 0.001
Male	1339 (52.7%)	1174 (50.8%)	997 (49.8%)	883 (49.3%)	616 (40.9%)	
Female	1203 (47.3%)	1135 (49.2%)	1004 (50.2%)	909 (50.7%)	891 (59.1%)	
Race/Ethnicity						< 0.001
Mexican American	395 (15.5%)	398 (17.2%)	323 (16.1%)	226 (12.6%)	117 (7.8%)	
Other Hispanic	291 (11.4%)	258 (11.2%)	191 (9.5%)	164 (9.2%)	110 (7.3%)	
Non-Hispanic White	714 (28.1%)	872 (37.8%)	916 (45.8%)	945 (52.7%)	970 (64.4%)	
Non-Hispanic Black	810 (31.9%)	496 (21.5%)	328 (16.4%)	245 (13.7%)	161 (10.7%)	
Other Race	332 (13.1%)	285 (12.3%)	243 (12.1%)	212 (11.8%)	149 (9.9%)	
Education categories						< 0.001
≤ High school	1315 (51.7%)	1088 (47.1%)	856 (42.8%)	690 (38.5%)	602 (39.9%)	
> high school	1227 (48.3%)	1221 (52.9%)	1145 (57.2%)	1102 (61.5%)	905 (60.1%)	
PIR (Poverty Income Ratio)						< 0.001
Low (0–1.56)	971 (42.5%)	815 (38.4%)	627 (34.2%)	515 (30.8%)	431 (30.6%)	
Middle (1.57–3.62)	691 (30.2%)	627 (29.6%)	550 (30.0%)	512 (30.7%)	480 (34.1%)	
High (3.63–5)	625 (27.3%)	679 (32.0%)	656 (35.8%)	643 (38.5%)	496 (35.3%)	
BMI (Body Mass Index, kg/m^2^)						< 0.001
< 24.9	794 (31.2%)	592 (25.6%)	529 (26.4%)	469 (26.2%)	335 (22.2%)	
25–29.9	856 (33.7%)	829 (35.9%)	697 (34.8%)	630 (35.2%)	515 (34.2%)	
≥ 30	892 (35.1%)	888 (38.5%)	775 (38.7%)	693 (38.7%)	657 (43.6%)	
Smoking consumption						< 0.001
Never	1305 (51.3%)	1298 (56.2%)	1156 (57.8%)	1063 (59.3%)	879 (58.3%)	
Former	527 (20.7%)	541 (23.4%)	506 (25.3%)	485 (27.1%)	484 (32.1%)	
Current	710 (27.9%)	470 (20.4%)	339 (16.9%)	244 (13.6%)	144 (9.6%)	
Alcohol consumption						< 0.001
Never	315 (12.4%)	276 (12.0%)	241 (12.0%)	234 (13.1%)	219 (14.5%)	
Moderate	767 (30.2%)	739 (32.0%)	695 (34.7%)	664 (37.1%)	571 (37.9%)	
Heavy	532 (20.9%)	508 (22.0%)	419 (20.9%)	342 (19.1%)	267 (17.7%)	
Binge	298 (11.7%)	288 (12.5%)	208 (10.4%)	150 (8.4%)	75 (5.0%)	
Not recorded	630 (24.8%)	498 (21.6%)	438 (21.9%)	402 (22.4%)	375 (24.9%)	
General health condition						< 0.001
Excellent/Very good/Good	1758(69.2%)	1663 (72.0%)	1472 (73.6%)	1345 (75.1%)	1116 (74.1%)	
Fair/Poor	583 (22.9%)	489 (21.2%)	409 (20.4%)	339 (18.9%)	316 (21.0%)	
Not recorded	201 (7.9%)	157 (6.8%)	120 (6.0%)	108 (6.0%)	75 (5.0%)	
Diabetes						< 0.001
Yes	380 (14.9%)	390 (16.9%)	365 (18.2%)	358 (20.0%)	376 (25.0%)	
No	2162 (85.1%)	1919 (83.1%)	1636 (81.8%)	1434 (80.0%)	1131(75.0%)	
Mean PD (mm)	1.7 ± 0.7	1.6 ± 0.6	1.5 ± 0.6	1.5 ± 0.6	1.4 ± 0.5	< 0.001
Extent PD ≥ 4 mm (%)	3.9 ± 7.9	3.3 ± 7.7	2.6 ± 7.0	2.2 ± 5.8	1.7 ± 4.6	< 0.001
Mean CAL (mm)	2.0 ± 1.3	1.8 ± 1.1	1.8 ± 1.2	1.8 ± 1.1	1.8 ± 1.0	< 0.001
Extent CAL ≥ 3 mm (%)	16.6 ± 17.8	14.7 ± 16.7	14.1 ± 16.3	13.5 ± 15.7	13.3 ± 14.9	< 0.001
CDC/AAP case definition						< 0.001
No/mild periodontitis	1224 (48.2%)	1241 (53.7%)	1132 (56.6%)	1026 (57.3%)	859 (57.0%)	
Moderate/severe periodontitis	1318 (51.8%)	1068 (46.3%)	869 (43.4%)	766 (42.7%)	648 (43.0%)	

*PD* periodontal pockets probing depth, *CAL* loss of clinical attachment, *CDC* Centers for Disease Control and Prevention, *AAP* American Academy of Periodontology

### The association of RBC folate with moderate/severe periodontitis

The study used logistic regression models to determine the relationship between RBC folate and moderate/severe periodontitis in different models (Table 2). The data showed a significantly skewed distribution when RBC folate was used as a continuous variable (normality test *p* < 0.0001), so we performed a log2 transformation [32]. When RBC folate was analyzed as a continuous variable, there was a statistical association between RBC folate (log2 transformation) and moderate/severe periodontitis after adjusting all confounders (OR 0.79, 95% CI 0.72–0.85, *p* = 0.0001). This association remained when RBC folate was assessed as five groups in different models. In model 2, the periodontitis risk of Q2-Q5 was significantly reduced compared with Q1, and the trend of protection was more pronounced as the concentration of RBC folate increased (Q2: OR 0.88 95% CI 0.77–1.01, *p* = 0.0714; Q3: OR 0.83, 95% CI 0.72–0.96, *p* = 0.0106; Q4: OR 0.77, 95% CI 0.67–0.90, *p* = 0.0009; Q5: OR 0.65, 95% CI 0.56–0.77, *p* < 0.0001). The risk of moderate/severe periodontitis in Q2, Q3, Q4, and Q5 was reduced by 12%, 17%, 23% and 45% respectively. This suggested that there may have been a nonlinear relationship between the two before and that the trend test (*p* < 0.0001) indicated that the results were relatively robust.

**Table 2:** Multivariable-adjusted odds ratios and 95% confidence intervals (*p-value*) of RBC folate levels associated with moderate/severe periodontitis in different models

Exposure	Unadjusted	*p*	Model 1	*p*	Model 2	*p*
Red blood cell folate (Log2 transform)	0.80 (0.75, 0.85)	< 0.0001	0.70 (0.65, 0.76)	0.0002	0.79 (0.72, 0.85)	< 0.0001
Q1(Reference)	1.0		1.0		1.0	
Q2	0.80 (0.71, 0.89)	0.0001	0.79 (0.70, 0.90)	0.0003	0.88 (0.77, 1.01)	0.0741
Q3	0.71 (0.63, 0.80)	< 0.0001	0.69 (0.61, 0.79)	< 0.0001	0.83 (0.72, 0.96)	0.0106
Q4	0.69 (0.61, 0.78)	< 0.0001	0.63 (0.55, 0.72)	< 0.0001	0.77 (0.67, 0.90)	0.0009
Q5	0.70 (0.62, 0.80)	< 0.0001	0.56 (0.48, 0.64)	< 0.0001	0.65 (0.56, 0.77)	< 0.0001
P for trend	< 0.0001		< 0.0001		< 0.0001	

Unadjusted model

Model 1 adjusted for Sex, Age, Race

Model 2 adjusted for Sex, Age, Race, Educational level, PIR, BMI, Alcohol consumption, Smoking consumption, General health condition, and diabetes

*RBC* Red blood cell folate, *PIR* Poverty Income Ratio, *BMI* Body Mass Index, kg/m^2^

**Fig. 3:**
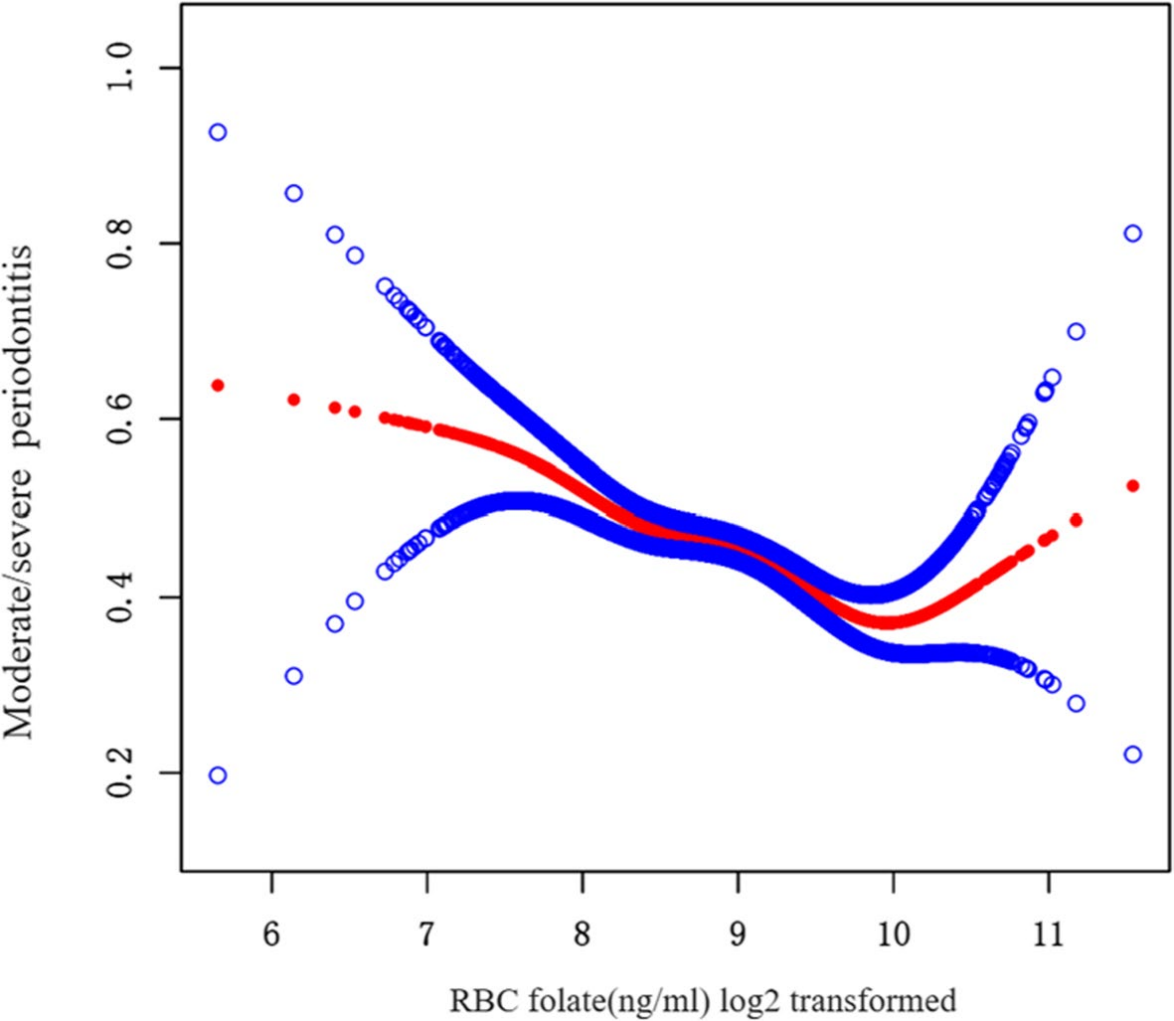
Dose–response relationship between RBC folate levels (Log2 transformation) and moderate/severe periodontitis: there was a nonlinear threshold effect (in generalized additive models). To adjust for the following parameters: sex, age(smooth), race, educational level, PIR, BMI, alcohol consumption, smoking consumption, general health condition, and diabetes

The restricted cubic spline (RCS) plots (Supplementary Fig. 1) and curve-fitting analysis (Fig. 3) showed a nonlinear relationship between RBC folate and periodontitis (*p* for nonlinear < 0.001), with a clear inflection point, which suggested that once RBC folate concentration exceeds the inflection point then its effect on periodontitis changes (threshold effect). Red represents curve fitting and blue lines represent confidence intervals. Meanwhile, the linear regression model and the two-segment linear regression model were compared, and the log-likelihood ratio test was *p* = 0.016. This indicates that a two-segment linear regression model should be used to fit the model. The infection point was calculated to be 9.88 (log2 transformation) by a two-segment linear regression model and recursive algorithm (Table 3). When the RBC folate (log2 transformation) was < 9.88, the risk of periodontitis was reduced by 25% for each additional unit of RBC folate (OR = 0.75, 95% CI = 0.69–0.82, *p* < 0.0001). Increased RBC folate concentration (> 9.88) was a risk factor for moderate/severe periodontitis (OR = 1.60), although it was not statistically significant (Table 3).

**Table 3:** Threshold effect analysis of the RBC folate levels (Log2 transformation) and periodontitis using piece-wise logistic regression

Outcome	moderate/severe periodontitis	
	OR (95% CI)	*p-*value
Fitting by weighted logistic regression model	0.79 (0.72, 0.85)	< 0.0001
Fitting by weighted two-piecewise linear logistic mode		
Inflection point		
< 9.88	0.75 (0.69, 0.82)	< 0.0001
> 9.88	1.60 (0.89, 2.86)	0.1146
Log-likelihood ratio test	0.016	

### Subgroup analysis

**Fig. 4:**
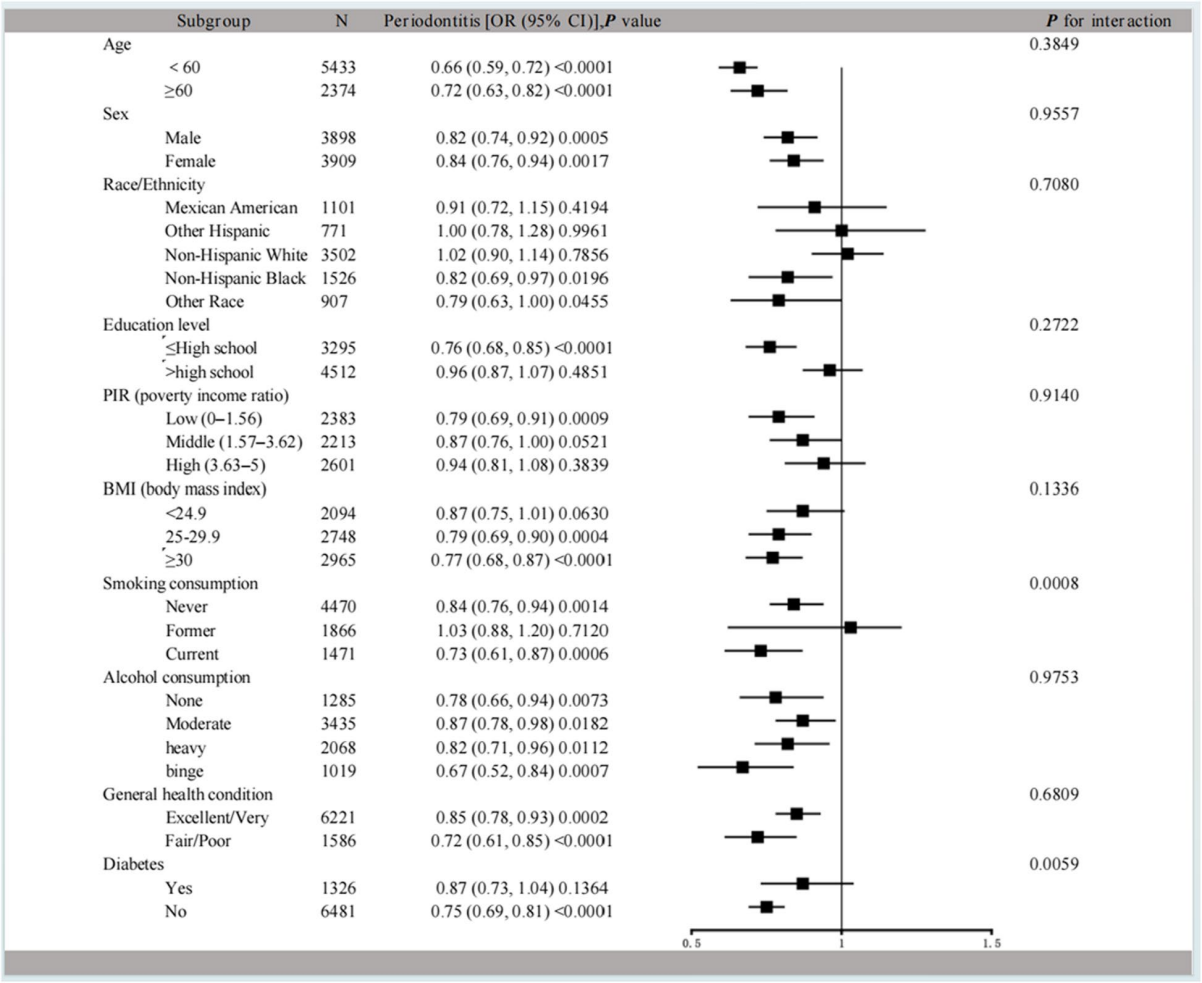
Forest Map-Subgroup analyses of the effect of RBC folate concentration on moderate/severe periodontitis

Adjusting for all confounders (model 2), the results of the subgroup analyses showed that the RBC folate was similarly associated in most subpopulations (Fig. 4). The influence of RBC folate on periodontitis is more significant in the “Never/Current” smokers (“Never”: OR 0.84, 95% CI 0.76–0.94, *p* = 0.0014; “Former”: OR 1.03, 95% CI 0.88–1.20, *p* = 0.7120; “Current”: OR 0.73, 95% CI 0.61–0.87, *p* = 0.0006; *p* for interaction = 0.0008). The effect of RBC folate on periodontitis was more pronounced in participants without diabetes (Yes: OR 0.87, 95% CI 0.73–1.04, *p* = 0.1364; No: OR 0.75, 95% CI 0.69–0.81, *p* < 0.0001; *p* for interaction = 0.0059). Other confounders had no statistically significant interaction on the association between RBC folate and moderate/severe periodontitis.

## Discussion

The study was the first to describe the relationship between RBC folate and periodontitis. As NHANES collected information on oral periodontal tissue health from 2009 to 2014, the study included 10,151 participants. After adjusting other confounders, RBC folate was negatively associated with moderate/severe periodontitis. Higher RBC folate was associated with a lower risk of moderate/severe periodontitis than lower RBC folate. In most subgroup populations, RBC folate remains a protective factor for periodontitis (Fig. 4). “Current” smokers have a higher likelihood of developing periodontitis relative to those smoking “never”. Smoking has been verified to affect periodontal health [42]. Reduced leukocyte chemotaxis, decreased immunoglobulin production, and a stronger inflammatory response with an increased release of potentially tissue-destructive substances (e.g., reactive oxygen species, collagenase, serine proteases, and proinflammatory cytokines) are among the changes that tobacco smoking appears to induce [43]. Interestingly, it was proved that the negative association between RBC folate and periodontitis is more obvious in individuals without diabetes. More investigation may be required to clarify the underlying causes.

This study is consistent with previous cross-sectional studies on the relationship between folate and oral periodontal health [15, 16], and further expands our understanding of this relationship. In a study of only 879 participants, low serum folate levels were associated with periodontal disease in older adults, and the diagnostic criteria for periodontal disease included periodontitis and gingivitis [22]. The difference is that this study used CDC/AAP criteria to refine the diagnosis of periodontitis and limit the scope of the disease [5]. This is one of the strengths of this study, which provides a more accurate and granular oral health assessment. In addition, the study used larger samples of American adults. However, our study did not find a significant difference after stratified age (< 60, >  = 60 years).

This study used the AAP/CDC periodontitis case definition, which was proposed by Eke et al. in 2012 and was applied as a global standard for epidemiologic studies of periodontal disease [5]. A revised classification of periodontal diseases with multidimensional staging and grading capabilities was released by the European Federation of Periodontology/American Academy of Periodontology (EFP/AAP) in 2018 [44]. Based on severity, complexity, and the rate of disease progression, the 2018 EFP/AAP categorization of periodontal disease is split into 4 stages and 3 grades. Interdental CAL was used as a staging criterion [44]. However, non-periodontitis causes result in CAL, such as traumatic gingival recession, caries reaching the cervical area of the tooth, and endodontic lesion drainage through the marginal periodontal tissue, etc. [44]. Therefore, using the 2018 (EFP/AAP) classification may overestimate the prevalence of periodontitis due to the source of CAL not being specified in the NHANES database” [34].

The study used curve fitting to elucidate the nonlinear relationship between RBC folate concentration and periodontitis. Previous studies reported that inadequate folate intake is associated with the severity of periodontitis [16]. Systemic intake of folate to assist in periodontal cleaning and root planning can improve the clinical and biochemical effects of periodontitis [16]. Using multivariate analysis, M Esaki et al. found that low dietary folate intake was associated with bleeding gums in nonsmoking adult Japanese [45]. More than 80 countries worldwide have made it mandatory to supplement folate in cereals such as flour, and corn flour [46]. We hypothesized that there are two possible mechanisms to explain the reduction of the risk of periodontitis by folate. On the one hand, a decrease in folate levels may negatively affect the immune system, increasing inflammation and affecting the health of periodontal tissues [47]. On the other hand, folate is involved in DNA methylation and synthesis, protein and RNA synthesis, and promotes the formation and repair of periodontal tissue [10].

Notably, this study found that when the concentration of red blood cell folate was higher than 942.27 ng/ml, it turned into a risk factor for moderate/severe periodontitis, although it was not statistically significant. In the dose range of folic acid of 4200-400ug, folic acid is absorbed and converted to the active form of 5-methyltetrahydrofolate, reaching a saturated state [48]. That is, the body is unable to metabolize and utilize high doses of folate. Folate in natural foods, folate in supplements, and the state's mandatory addition of folate to cereals may cause elevated serum folate and RBC folate. Studies have reported that high physiological folate concentrations and folate overload in certain populations may increase the risk of impaired brain development and cancer during embryogenesis [49]. Ulrich CM et al. believe that when folate levels in the human body are higher, tumor cells can synthesize nucleotides more efficiently, thereby increasing the replication speed of DNA and further promoting tumor growth and spread [50]. High folate intake may lead to dihydrofolate accumulation, affecting DNA methylation. Regular checks for RBC folate concentrations (long-term markers of folate) help us properly control folate intake.

Limitations: however, the study had certain limitations that merited consideration. Firstly, the study was a cross-sectional study, which was unable to establish the causality of RBC folate concentration on periodontitis. Secondly, the effects of residual confounding cannot be completely ruled out in this study. Thirdly, it is worth noting that RBC folate showed a skewed distribution as a continuous variable during the curve fitting and threshold effect analyses, and therefore we performed a log2 transformation, which could be slightly biased from the true nonlinear relationship. We will continue to follow up on the NHANES updates and perform relevant analyses to verify the robustness of the results, which will serve as the beginning of our next study. Additionally, we are looking forward to further research to elucidate the mechanisms linking the two to help develop more effective prevention and treatment strategies that will improve people's oral health.

## Conclusion

This cross-sectional study revealed a negative relationship between the RBC folate and moderate/severe periodontitis within a certain threshold range. Dentists and policymakers should pay closer attention to oral hygiene and health care for people with low or high RBC folate levels. Further causal and longitudinal research mechanisms are needed to validate our findings.

### Supplementary Information

Supplementary Material 1.

## Data Availability

The datasets and related information (excluding personally identifiable information) used in this study can be downloaded from the NHANES database(https://www.cdc.gov/nchs/nhanes) for free.
