# Supplementary material for: Red blood cell folate level is associated with periodontitis in American adults: results from the NHANES 2009–2014
Source: BMC Oral Health. 2024 Jul 21;24:825. doi: 10.1186/s12903-024-04599-7 (PMC11265046; doi:10.1186/s12903-024-04599-7)
Supplement: Supplementary file 1 — Supplementary Material 1. [file 12903_2024_4599_MOESM1_ESM.pdf]

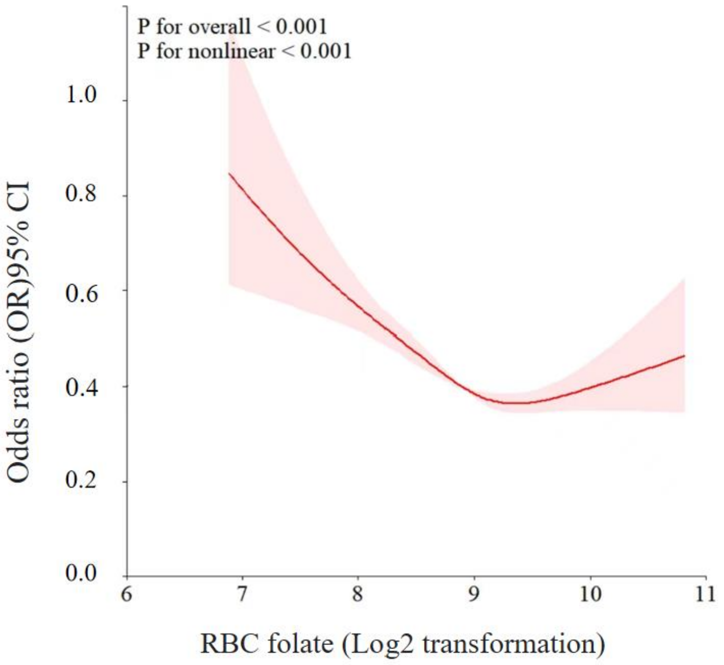

Supplementary figure 1 The restricted cubic spline (RCS) of

RBC folate levels (Log2 transformation) and moderate/severe periodontitis.
